# Periventricular Leukomalacia Following Bowel Resection for Necrotizing Enterocolitis in a Premature Neonate

**DOI:** 10.7759/cureus.45865

**Published:** 2023-09-24

**Authors:** Timothy B Williams, Sonia Kapoor, Carleene Bryan

**Affiliations:** 1 Osteopathic Medicine, Dr. Kiran C. Patel College of Osteopathic Medicine, Nova Southeastern University, Fort Lauderdale, USA; 2 Neonatology, Wellington Regional Medical Center, Wellington, USA

**Keywords:** pediatrics and neonatology, neonatal intensive care unit (nicu), pediatric gi surgery, periventricular leukomalacia, necrotizing enterocolitis, premature neonate

## Abstract

Necrotizing enterocolitis (NEC) and periventricular leukomalacia (PVL) are relatively common conditions in premature infants with low birth weight (VLBW). However, in the current literature, there are limited case reports of patients with concomitant NEC and PVL. We report a case of a premature female born at a gestational age of 25 weeks and five days who developed cystic intracranial lesions after emergent bowel resection due to NEC. Transcranial ultrasound and magnetic resonance imaging confirmed the presence of cystic PVL in the right middle cerebral artery distribution. Several observational studies note the association between spontaneous intestinal perforation, surgical NEC, and the presence of cystic PVL. When infants are unresponsive to medical management for NEC, exploratory laparotomy with resection of the necrotic or perforated intestine is indicated. However, infants treated surgically have poorer neurodevelopmental outcomes than those with medical therapy. Pathogenesis of neurodevelopmental impairment in preterm infants undergoing surgery involves dysfunctional cerebrovascular autoregulation (CAR), which is associated with harmful changes in cerebral perfusion that lead to neuronal injury. Ill preterm infants, such as those with NEC, cannot regulate cerebral perfusion appropriately, and impaired CAR may be present in more than half the preterm infants during laparotomy. Impaired CAR leads to poor cerebral perfusion that potentiates neuronal injury, such as PVL. This case also brings awareness to the need for adherence to screening practices for white matter injury in critical NICU patients through cost-effective transcranial ultrasound.

## Introduction

Necrotizing enterocolitis (NEC) is a gastrointestinal emergency in newborns defined by intestinal inflammation that leads to bacterial invasion and ischemic necrosis of the intestinal mucosa [1]. More than 90% of NEC cases occur in very low birth weight (VLBW) infants born before 32 weeks of gestation with a birth weight under 1500 g [1]. The clinical presentation of NEC is non-specific and varies among infants. It includes a sudden change in feeding tolerance, bilious emesis, diarrhea, hematochezia, lethargy, abdominal distension, decreased bowel sounds, and respiratory failure [1]. Along with clinical findings, hallmark features used to confirm the diagnosis of NEC on abdominal X-rays are an abnormal gas pattern with dilated bowel loops, pneumatosis intestinalis, and portal venous air [1]. Initial treatment of NEC involves cessation of all enteral feedings, decompressing the dilated bowels with a nasogastric tube, and beginning broad-spectrum IV antibiotics. Between 5 and 10% of preterm infants will develop NEC, and approximately 30-50% of infants with NEC will progress to surgical management [2].

Pathogenesis of neurodevelopmental impairment in preterm infants undergoing surgery involves dysfunctional cerebrovascular autoregulation (CAR), which is associated with variations in cerebral perfusion [3,4]. Harmful changes in cerebral perfusion can lead to neuronal injury, including periventricular leukomalacia, due to cerebral ischemia [3,4]. More significantly, ill preterm infants, such as those with NEC, cannot regulate cerebral perfusion appropriately [3,4]. Kuik et al. showed impaired CAR was present in over half of the preterm infants during laparotomy [3]. While CAR is impaired during laparotomy, it is maintained pre and postoperatively. Furthermore, Stolwijk et al. demonstrated parenchymal lesions, including cerebral punctate lesions, on magnetic resonance imaging (MRI) in 75% of preterm infants after surgery [4].

Periventricular leukomalacia (PVL) is a neurological condition following injury to the brain's white matter [5,6]. In PVL, the death of white matter is characterized by liquefactive necrosis of brain tissue [5,6]. On neonatal examination, PVL may not present with prominent characteristics of neurological defects [7,8]. However, in severe cases, hypotonia, poor feeding, lethargy, apnea, and focal neurological deficits can present [7,8]. Although PVL can affect infants during fetal development, premature infants are at the highest risk, especially those with a birth weight of less than 1500 g and born between 23 and 30 weeks gestation [5]. The prevalence of PVL in infants under 32 and 37 weeks is 27.4% and 7.3%, respectively [9]. Other risk factors include hypoxia-ischemia (HI) insult, intrauterine infection, and chorioamnionitis, which all decrease the proper regulation of cerebral blood flow [3-5]. PVL is diagnosed with transcranial ultrasound (US) or magnetic resonance imaging (MRI), which can demonstrate focal cystic lesions after white matter injury (WMI) [5]. The 2020 American Association of Pediatrics screening guidelines recommend screening premature infants using cranial ultrasound, first at 7-10 days after birth, with subsequent imaging at four to six weeks after birth [10]. Focal cystic lesions are the most common, with a prevalence of 39.6% in premature infants younger than 28 weeks [9]. They appear as hypoechoic lesions involving the basal ganglia and periventricular matter on ultrasound [7]. Meanwhile, hemorrhagic causes involve the posterior internal capsule, resulting in lower extremity manifestations and progression spastic diplegia [7]. The prognosis of PVL depends on the extent of damage to the brain tissue, and its presentation can range from mild neurological deficits to severe consequences such as cerebral palsy [5,11-13].

## Case presentation

**Figure 1:**
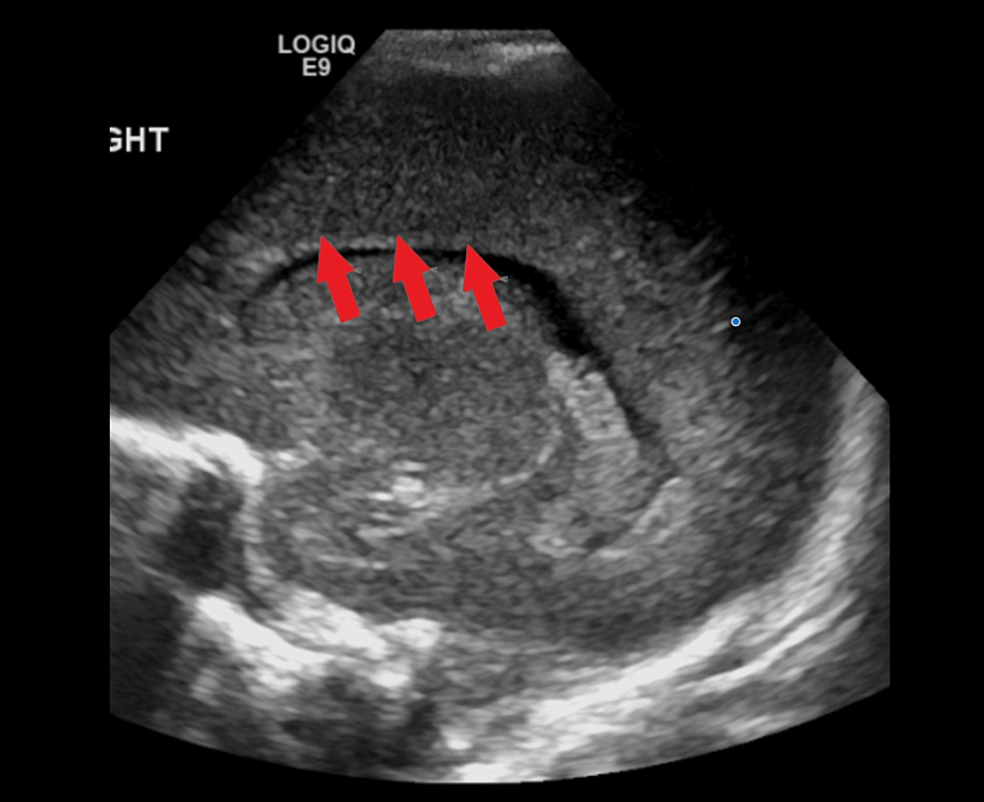
Initial transcranial ultrasound in the sagittal plane on day five showing the right hemisphere of the brain with normal findings.

A premature female patient was born at a gestational age of 25 weeks and five days to a 37-year-old G6P4 mother who developed cystic intracranial lesions following a bowel resection due to NEC. Pregnancy was complicated by preterm premature rupture of membranes (PPROM) and non-reassuring fetal heart rate (FHR). The patient was born via an emergent cesarean section under spinal anesthesia. APGAR scores were three at 1 minute, six at 5 minutes, and eight at 10 minutes. Positive pressure ventilation (PPV) was given, followed by intubation due to intermittent apnea and fluctuating heart rate (HR). The patient was transferred to the neonatal intensive care unit (NICU), where she was diagnosed with apnea of prematurity and placed on high-frequency oscillatory ventilation (HFOV). On admission at one day old, the arterial blood gas demonstrated metabolic acidosis with a pH of 7.26, pCO_2_ of 45, and pHCO_3_- of 20.2. The closest arterial blood gas to the initial ultrasound was performed at five days old. It demonstrated metabolic acidosis with a pH of 7.32, pCO_2_ of 40, and HCO_3_- of 20.6. Initial head ultrasound at seven days old showed no intraventricular hemorrhage (IVH) or cystic lesions (Figure 1). Empiric IV ampicillin at 345 mg/kg/day twice daily and IV gentamicin at 2 mg/kg/day twice daily was initiated pending blood cultures and completed over the course of seven days. Total parenteral nutrition needs were met accordingly, and the patient was transitioned to enteral feeding via nasogastric tube on day three.

**Figure 2:**
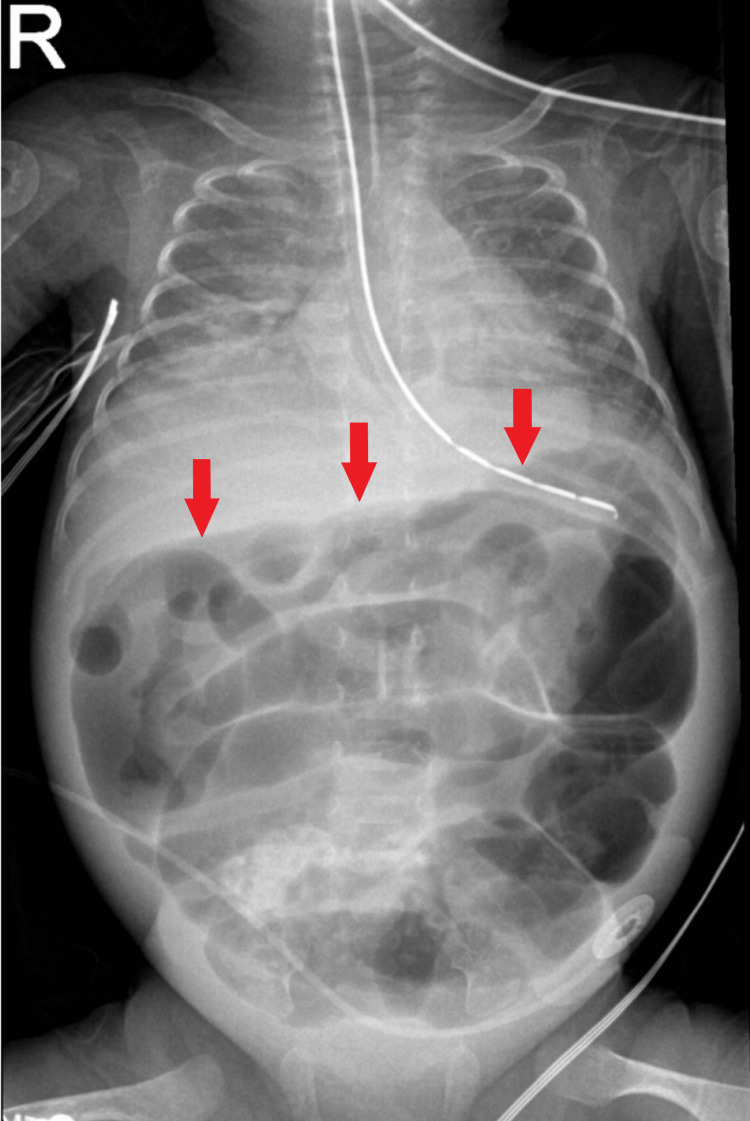
Antero-posterior infant X-ray demonstrating gaseous distention of bowel loops without pneumatosis or portal vein gas on day 47.

Upon day 47, the patient demonstrated increasing abdominal distention and progressive lethargy over 24-48 hours. On day 48, primary peritoneal drainage was attempted at the bedside. X-ray of the chest and abdomen on day 49 revealed moderate gaseous distension and favored ileus without any definite pneumatosis or portal venous gas (Figure 2). Stage III NEC was diagnosed, given the progression of bowel dilation, skin erythema of the abdominal wall, consumptive thrombocytopenia, and critical status of the patient.

From day 51 to 52, platelets decreased from 61,000 to 17,000, attributed to consumptive coagulopathy secondary to NEC. Consequently, platelet transfusion was initiated to increase platelets to 50,000 for surgery. On day 55, the patient presented with platelet levels of 70,000 and tolerated respiratory support with synchronized intermittent mandatory ventilation (SIMV). On day 56, capillary blood gas demonstrated metabolic alkalosis with a pH of 7.48, pCO_2 _of 46, and HCO_3_- of 34.3. With a final transfusion, the patient underwent surgery on day 56 with platelet levels of 94,000.

On day 56, the patient underwent exploratory laparotomy and bowel resection with ileostomy under general anesthesia due to the severity of intestinal inflammation with a high risk of perforation and persistent small bowel loops consistent with NEC despite medical management for nine days. NEC was present in all distal small bowels, from the ligament of Treitz to 2 cm from the ileocecal valve. A total of 30 cm of necrotic bowel was resected, and an ileostomy was formed. The surgical course was uncomplicated, with an estimated blood loss of 10 mL. Pathological evaluation of the resected small bowel described completely necrotic portions of the small bowel with coagulative necrosis of the mucosa and underlying micro abscess formation, ischemic changes, and granuloma formation, consistent with necrotizing ileitis.

**Figure 3:**
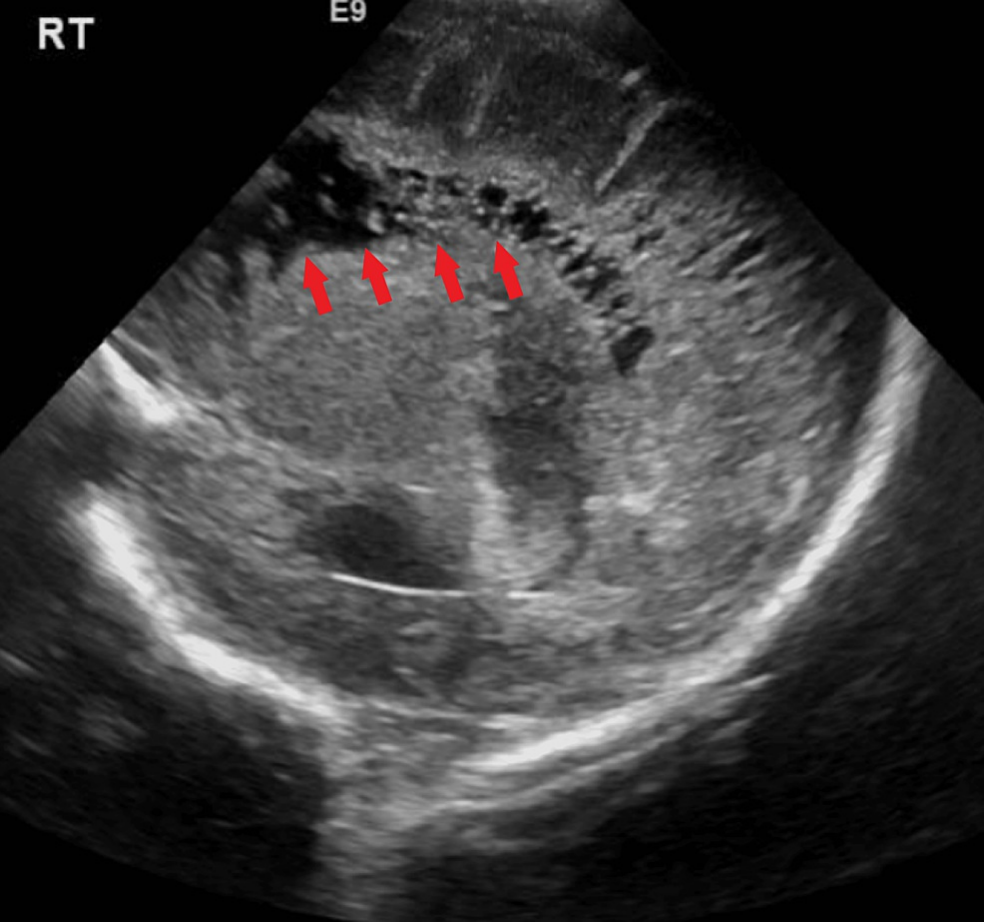
Transcranial ultrasound in the sagittal plane demonstrating cystic lesions in the distribution of the right MCA on postoperative day 21. MCA: middle cerebral artery Multiple bilateral cystic foci involving the periventricular white matter were noted.

**Figure 4:**
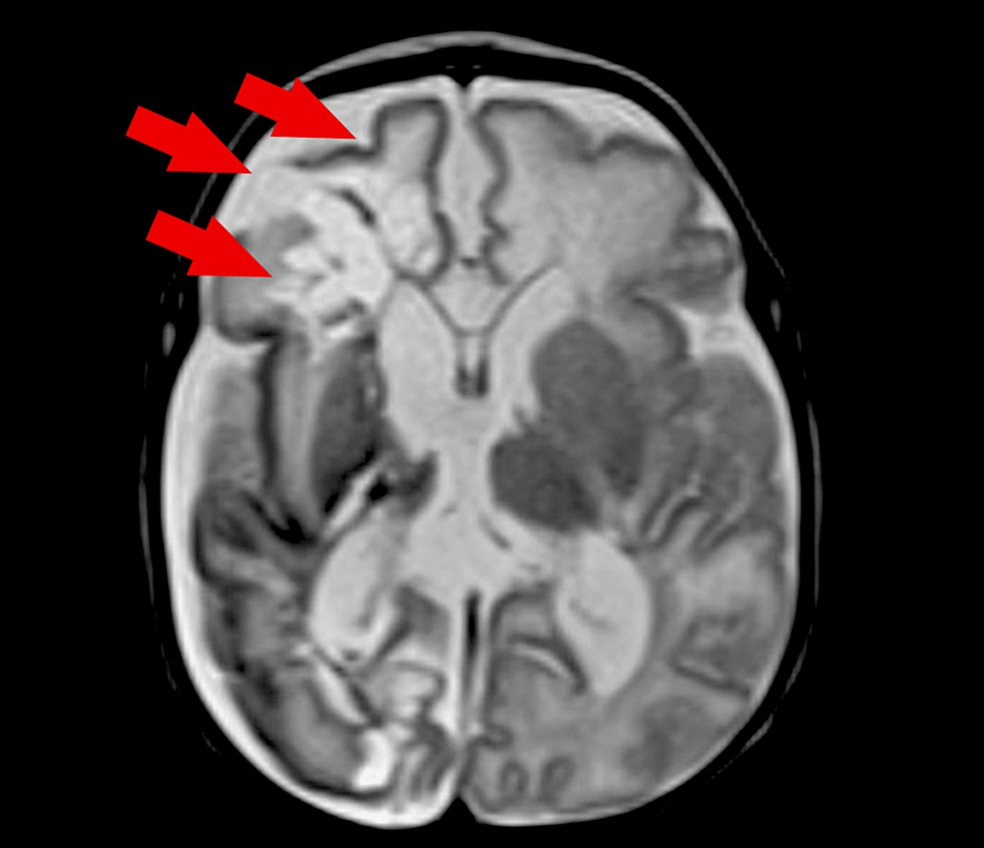
T2-weighted MRI in the transverse plane demonstrating bilateral cystic foci primarily in the distribution of the right MCA on postoperative day 21. MCA: middle cerebral artery

**Figure 5:**
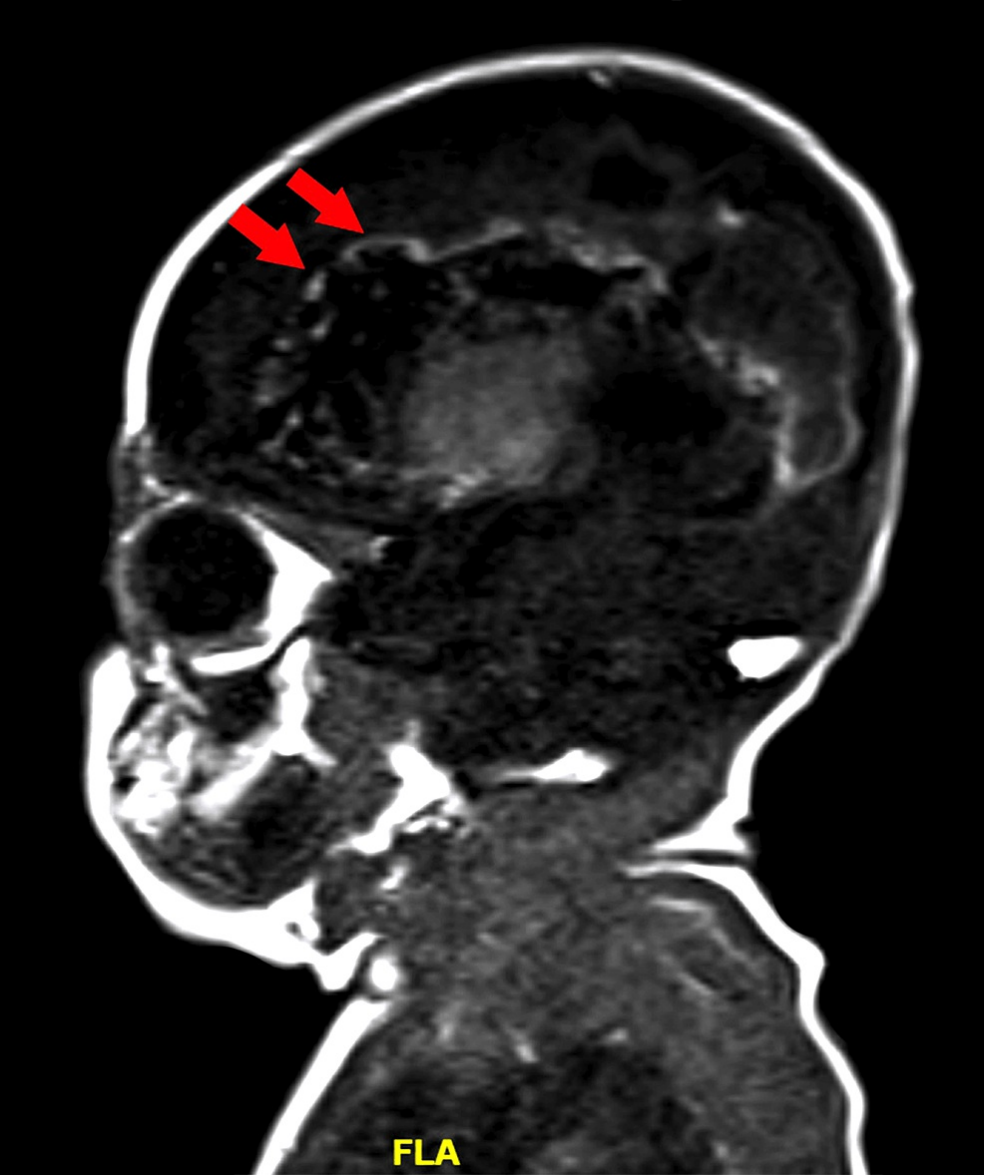
T1-weighted MRI in the sagittal plane demonstrating bilateral cystic foci, primarily in the distribution of the right MCA on postoperative day 21. MCA: middle cerebral artery

On return to the NICU, pain management and sedation were continued for eight days. The patient was not taking any anticoagulants. Antibiotics and pain management were discontinued, with no bacteriemia throughout the disease progression. On postoperative day 21, the patient presented with three episodes of jerking movements and myoclonic spasm, with no observable seizure activity on electroencephalogram (EEG), consistent with normal findings. On transcranial ultrasound, multiple bilateral cystic foci involving the periventricular white matter were present (Figure 3). Radiological impression was consistent with PVL involving the basal ganglia and a small right IVH. There was also mild, diffuse ventricular prominence with no overt hydrocephalus. Magnetic resonance imaging (MRI) without contrast confirmed the presence of bilateral cystic PVL and a significant focus of subacute ischemia in the right middle cerebral artery (MCA) distribution (Figures 4, 5). Myoclonic jerks on physical examination represented evidence of motor deficit due to ischemic injury. The patient was transferred to a Level II NICU and pediatrics hospital for further management per parental request.

## Discussion

PVL is a form of WMI characterized by the demyelination of pre-myelinating oligodendrocytes (pre-OL) due to hypoxia-ischemic insult. Inflammatory disease and infection significantly contribute to the development of HI insult and the likelihood of cystic focal PVL. The overgrowth of Gram-positive and Gram-negative anaerobes accompanies NEC [14]. Activation of toll-like receptor 4 (TLR-4) by bacterial lipopolysaccharide (LPS) and other pathogen-associated molecular patterns (PAMPs) can initiate various systemic proinflammatory cytokine pathways, such as mitogen-activated protein kinase (MAPK) and nuclear factor kappa-B (NF-kB) [15,16]. The activation of these pathways is a known contributor to microglial activation and glutamate excitotoxicity in the central nervous system, leading to the generation of reactive oxygen species (ROS) and reactive nitrogen species (RNS) [5,11,15]. The subsequent death of pre-OL, axonal injury, subplate neuronal injury, and GABAergic neuron injury results in impaired accumulation of myelin, reduced cortical development, and loss of neuronal plasticity [12,13,17]. These changes are observable as white matter loss, cystic necrosis, and ventriculomegaly - the hallmark characteristics of PVL [12,13,17].

Children with PVL should receive regular medical screening examinations relevant to the range of symptoms they may be experiencing [18]. These symptoms range from physical to mental development, and children should be assessed regularly by a developmental specialist. For children who develop movement disorders, such as a walking disorder, treatments such as short leg braces, wheelchairs, botulinum toxins, and Achilles tendon extensions have been utilized [19]. In addition, various antiepileptics play a role in seizure management [19]. Other forms of therapy for children may include speech, occupational, and physical therapies [18]. Although there is no specific treatment for PVL, a supportive and symptomatic approach to patient care should be targeted.

This report presents a case involving the development of PVL following NEC and necessary surgical intervention in a VLBW premature female infant. Numerous studies demonstrate a clear association between NEC and PVL. A systematic review and meta-analysis by Matei et al. demonstrated a 43% incidence of neurodevelopmental impairment in surgically treated NEC patients [20]. Moreover, an 11% incidence of PVL was noted in premature NEC patients [20]. The timeline for the development of cystic lesions in this patient corresponds to the worsening NEC, a known cause of dysfunctional cerebral autoregulation and hypoxia-ischemia (HI) insults.

## Conclusions

This study describes the diagnosis of PVL, a severe neurological disease involving WMI, in a premature female patient with cystic intracranial lesions on MRI following a bowel resection with an ileostomy due to NEC. PVL is most common in premature babies, those who experience a hypoxia-ischemia injury or other insult during gestation or delivery. Although there is no definitive treatment for PVL, strong adherence to recommended screening guidelines, such as those provided by the American Association of Pediatrics, can aid in early diagnosis of PVL and rapid progression to appropriate management.
